# Porcine Circovirus Type 3 in Pig Farms Experiencing Diarrhea in Jiangxi, China: Prevalence, Genome Sequence and Pathogenicity

**DOI:** 10.3390/ani10122324

**Published:** 2020-12-07

**Authors:** Fanfan Zhang, Weifeng Yuan, Zhiquan Li, Yuhan Zhang, Xiuxiu Zeng, Min Zhao, Yu Ye, Zhen Ding, Houjun He, Qiong Wu, Deping Song, Yuxin Tang

**Affiliations:** 1 Department of Preventive Veterinary Medicine, College of Animal Science and Technology, Jiangxi Agricultural University, Nanchang 330045, China; zfanfan0816@163.com (F.Z.); ywf519@outlook.com (W.Y.); lizhiquan2017@outlook.com (Z.L.); zyhsh47@163.com (Y.Z.); zxx_3625@163.com (X.Z.); zhaomin202012@163.com (M.Z.); yy6157832@163.com (Y.Y.); dingzhenhuz@163.com (Z.D.); hehoujun@163.com (H.H.); lbls2005@sina.com (Q.W.); tang53ster@gmail.com (Y.T.); 2 Key Laboratory for Animal Health of Jiangxi Province, Nanchang 330045, China; 3 Institute of Animal Husbandry and Veterinary Medicine, Jiangxi Academy of Agricultural Sciences, Nanchang 330200, China; 4 Jiangxi Engineering Research Center for Animal Health Products, Jiangxi Agricultural University, Nanchang 330045, China

**Keywords:** porcine circovirus 3, diarrhea, prevalence, genome, pathogenicity

## Abstract

**Simple Summary:**

Porcine circovirus 3 (PCV3) is a new species of PCV that was associated with porcine dermatitis and nephropathy syndrome (PDNS), respiratory disease, cardiac and multisystem inflammation in nursery and finishing pigs, and reproductive failure problems, including abortion, mummified fetuses, and stillbirth in sows. To date, the reports on the PCV3 present in diarrhea pigs are limited and the genetic characteristics of PCV3 from diarrheal pigs and pathogenicity on pigs were inconsistent. This study aimed to investigate the prevalence of PCV3 in pigs with/without diarrhea, to analyze the genome sequence of PCV3 from diarrheal pigs, and to inquire into the associated pathogenicity of PCV3 to piglets experimentally infected with PCV3-positive intestinal contents. The results demonstrated that PCV3 was widely circulating in diarrheal suckling and weaned piglets. Clinical signs, gross lesions, and histological changes were observed in suckling piglets inoculated with PCV3. The complete genome of a PCV3a strain was determined and two mutations (V24A and K27R) were present when compared with PCV3b strains. The findings of this study increase the knowledge of the epidemiology, viral genetics, pathogenicity, and pathogenesis of PCV3.

**Abstract:**

Porcine circovirus 3 (PCV3) infections have been reported in different clinical presentations. However, the prevalence and pathogenicity of PCV3 associated with diarrhea in piglets have been limited. Herein, we present an investigation and genome analyses of PCV3 in piglets experiencing diarrhea, and observed clinical signs, gross lesions, and histological changes in pigs negative for all known pathogens associated with diarrhea but positive for PCV3 alone. Among the feces (*n* = 141) tested, 16.31% (23/141) were positive for PCV3. Of which, 27.28% (15/55) and 14.29% (5/35) were present in diarrheal samples from suckling and weaned piglets, respectively. Moderate to severe atrophic villi was confined in duodenum, jejunum, and ileum, and significantly decreased average heights of villi, and the depths of crypt were observed in PCV3-infected piglets. The complete genome of a representative strain of PCV3, designated as JX/CH/2018, was determined. Multialignment analysis indicated that JX/CH/2018 had 97.7–99.7% nucleotide identity at the complete genome level, and 97.2–100% at the amino acid level of the capsid protein when compared with reference PCV3 strains. Phylogenetic analysis showed that the PCV3 strain identified in this study belonged to PCV3a lineage. The present study demonstrated that PCV3 is a common virus in diarrheal suckling and weaned piglets.

## 1. Introduction

Porcine circoviruses (PCVs) are traditionally divided into two major genotypes: porcine circovirus 1 (PCV 1) and porcine circovirus 2 (PCV 2). PCV1 is not associated with clinical disease. PCV2 is a ubiquitous pathogen that causes a broad range of diseases and syndromes, including postweaning multisystemic wasting syndrome (PMWS), porcine dermatitis and nephropathy syndrome (PDNS), respiratory and enteric disease, and reproductive failure [1,2,3]. In 2016, a new species of PCV was first recognized in pigs from a case of PDNS and reproductive failure in the USA via next generation sequencing (NGS) technology, and designated as porcine circovirus 3 (PCV3) [4]. Subsequently, PCV3 was found to be widely distributed in pigs around the world, and up to date, it has been reported in at least 14 countries, including the USA, Japan, China, South Korea, Russia, Brazil, Thailand, Poland, Sweden, Italy, Germany, Denmark, Spain, and the UK [5,6,7,8,9,10,11,12]. In China, PCV3 was first reported in Liaoning Province, Jiangxi Province and Chongqing city in 2017 [13]. Previous analyses on retrospective studies indicated that PCV3 existed in China as early as 1996 [14]. Until now, PCV3 has been detected in 26 provinces in China [15,16,17].

The entire genome of PCV3 is about 2 kb in length which includes three open reading frames (ORFs)—that is, ORF1, ORF2, and ORF3. ORF1 encodes a 296 amino acid (aa) replicase protein (Rep), ORF2 encodes a 214 aa capsid protein (Cap), and ORF3 encodes a putative protein containing 123 aa, the function of which is still unclear [18,19,20]. Clinically, this newly discovered virus was usually associated with PDNS, respiratory disease, cardiac and multisystem inflammation in nursery and finishing pigs, and reproductive failure problems, including abortion, mummified fetuses, and stillbirth in sows [4,15,21]. Additionally, several studies implied PCV3 infections were associated with diarrhea in suckling and weaned piglets, while whether PCV3 was a causative agent for diarrhea in pigs is still unknown [15,20,22].

To investigate the prevalence of PCV3 in piglets with diarrhea in Jiangxi, China, feces or intestinal contents from piglets with/without diarrhea were collected and tested for presence of PCV3; clinical signs, gross lesions, and histopathological changes of piglets infected exclusively by PCV3 were observed; molecular characteristics of PCV3 associated with diarrhea was also analyzed. The aim of this study was to provide novel epidemiology, genetic, and pathogenicity information of PCV3 associated with diarrhea.

## 2. Materials and Methods

### 2.1. Ethical Approval and Consent to Participate

This study was approved by the Animal Ethics Committee of Jiangxi Agricultural University, Nanchang, China (approved number: JXAU-LL-2018-0021). All procedures involving animals in this study were conducted according to the guidelines for the care and use of experimental animals established by the Ministry of Agriculture and Rural Affairs of the People’s Republic of China.

### 2.2. Sample Collection

Feces and/or intestinal contents from 55 diarrheal suckling piglets, 35 diarrheal weaned pigs, 30 non-diarrheal suckling pigs, and 21 non-diarrheal weaned pigs were collected from 5 pig farms experiencing diarrhea in Jiangxi, China. These samples were collected by veterinarians in these farms based on the standard procedures, and then immediately submitted to our laboratory for diagnosis. All samples were recorded and aliquoted into 5 mL eppendorf tubes and stored at −80 °C until tested.

### 2.3. Detection of PCV3 and other Related Pathogens

Total RNAs/DNAs were extracted using MiniBEST Viral RNA/DNA Extraction Kit (TaKaRa, Dalian, China) according to the manufacturer’s instructions and then were stored at −80 °C until used. To investigate whether PCV3 was associated within these cases, a pair of primers (PCV3-F: 5′-TGGTGCCGTAGAAATCTGTC-3′, and PCV3-R: 5′-GCCTAAACGAATGGGAAACT-3′ with a predicted product size of 408 bp) for a polymerase chain reaction (PCR) was initially designed based on the ORF2 gene of PCV3-US/SD2016 (GenBank accession no. KX966193), and then a PCR assay was established with the designed primers. The PCR amplification was executed under the following conditions: denaturation at 94 °C for 5 min, 36 cycles of 94 °C 30 s, 52 °C 30 s, 72 °C 30 s, and consequently with a final extension at 72 °C for 7 min. Expected PCR products were purified, cloned, and sequenced based on the standard procedures. PCV2 and the previous reported diarrhea associated viruses, including classical swine fever (CSFV), porcine pseudorabies (PRV), porcine reproductive and respiratory syndrome virus (PRRSV), porcine epidemic diarrhea virus (PEDV), porcine deltacoronavirus (PDCoV), transmissible gastroenteritis virus (TGEV), porcine rotavirus (PoRV), and severe acute-diarrhea syndrome coronavirus (SADS-CoV) were tested by PCR/RT-PCR assays developed in our laboratory (primer sequences present in Table S1). In addition, common enteropathogenic germs, including pathogenic *Escherichia coli*, *Salmonella*, *Clostridium perfringens* and *Clostridia difficile*, and protozoa, including *Cryptosporidium spp*, *Strongyloides spp*, *Isospora suis,* and *Eimeria spp* were also tested via bacterial isolation and identification with the standard protocols.

### 2.4. Complete Genome Determination and Characterization of PCV3

To determine the complete genome of PCV3 associated with diarrhea in suckling piglets in Jiangxi, China, two pairs of primers (PCV3-1F: 5′-TAGTATTACCCGGCACCTCGGAACC-3′, PCV3-1R: 5′-ACAGGTAAACGCCCTCGCATGTGGG-3′, predicted product size is 1257 bp; PCV3-2F: 5′-TGCACTTGTGTACAATTATTGCG-3, and PCV3-2R: 5′-ATCTTCAGGACACTCGTAGCACCAC-3′, predicted product size is 1075 bp) were designed based on a multiple alignment analysis of published sequences of PCV3 by using the MegAlign program of DNAStar *Lasergene* version 7.10 (DNAStar, Inc., Madison, WI, USA). Viral DNA was extracted from a sample solo-infected with PCV3. Subsequently, two overlapping fragments covering the entire genome sequence of PCV3 were amplified by an inverse PCR assay with Phanta Max Super-Fidelity DNA Polymerase (Vazyme, Nanjing, China). All PCR products were subject to gel-purification. After an A-tail was added on, each amplicon was then cloned into the PMD18-T Vector (Takara, Dalian, China) and sequenced. Sequences of PCV3 were assembled and annotated using the aforementioned DNAStar software. Phylogenetic analyses of PCV3 were conducted based on the nucleotide (nt) sequences of complete genome, and the aa sequences of cap protein, respectively, by using the neighbor-joining method of Molecular Evolutionary Genetics Analysis (MEGA) software (v6.0.2, https://www.megasoftware.net/).

### 2.5. Clinical Signs, Gross Lesions, and Histopathology

To observe clinical signs, gross lesions and enteric histopathology of pigs with diarrhea probably induced by PCV3, six 3-week-old piglets were orally inoculated with PCV3 sole-positive intestinal contents with 3.0 × 10^6.5^ genomic copy number of PCV3 in 5 mL volume for each piglet. After 7 days post-inoculation (dpi), clinical signs, gross lesions, and histopathology were observed based on the standard procedures. Briefly, the pigs were euthanized via intravenous injection with pentobarbital sodium. Subsequently, duodenum, jejunum, ileum, cecum, colon, and rectum were collected and fixed in 4% (vol/vol) neutral phosphate-buffered paraformaldehyde, then embedded in paraffin, sectioned into 5 μm segments, and stained with hematoxylin and eosin (H&E). Microscopic lesions were observed on a microscope NIS-Elements 3.2 (Nikon, Tokyo, Japan). The villus heights and crypt depths were measured according to a previously described method [23]. The ratio of villus height (VH) and crypt depth (CD) of each intestinal section was calculated and statistically analyzed by student’s *t* test.

### 2.6. Statistical Analysis

Statistical analysis was performed using SPSS software 25.0 (IBM Corporation, Armonk, New York, NY, USA). The data of this study were assessed for the normal (Gaussian) distribution by using the Shapiro–Wilk Test in SPSS before performing the statistical analysis. Statistical significance among the villus height, crypt depth, and villus/crypt ratio was determined using one-way analysis of variance (ANOVA) and significant differences among group means were determined using the least significant difference (LSD) test. Data are presented as the mean ± standard deviation (SD). A *p* value of < 0.05 was set as the statistically significant level.

## 3. Results

### 3.1. Prevalence of PCV3 and Co-Infections in Diarrheal and Non-Diarrheal Pigs

Among 141 samples tested, 23 (16.31%) were positive for PCV3, of which 15 (15/55, 27.28%) in diarrheal samples from suckling pigs, 5 (5/35, 14.29%) in diarrheal samples from weaned pigs, 2 (2/30, 6.67%) in non-diarrheal samples from suckling pigs, and 1 (1/21, 4.76%) in non-diarrheal samples from weaned pigs (Table 1). The PCV3-positive rate was much higher in diarrheal piglets (20/90, 22.22%) than that in non-diarrheal weaned pigs (3/51, 5.88%). Additionally, we also examined the co-infections in these samples. Of 55 diarrheal samples from suckling piglets, 17 (30.91%) were PCV2-positive and 5 (9.09%) were co-infected by PCV3 and PCV2, 5 (5.71%) samples from diarrheal weaned pigs were co-infected by PCV3 and PCV2, and 1 sample from non-diarrheal weaned pigs were positive for both PCV3 and PCV2. PEDV and PDCoV were found positive in 5 and 1 diarrhea samples, respectively. Among these pigs, nine samples from diarrheal suckling piglets were found solely positive for PCV3, but negative for PCV2 and all other diarrhea associated viruses, including CSFV, PRV, PRRSV, TGEV, PoRV, PDCoV, SADS-CoV, and any other known bacterial pathogens, including pathogenic *Escherichia coli*, *Salmonella*, *Clostridium perfringens* and so on.

### 3.2. Complete Genome Sequencing and Genetic Analysis of PCV3

**Figure 1:**
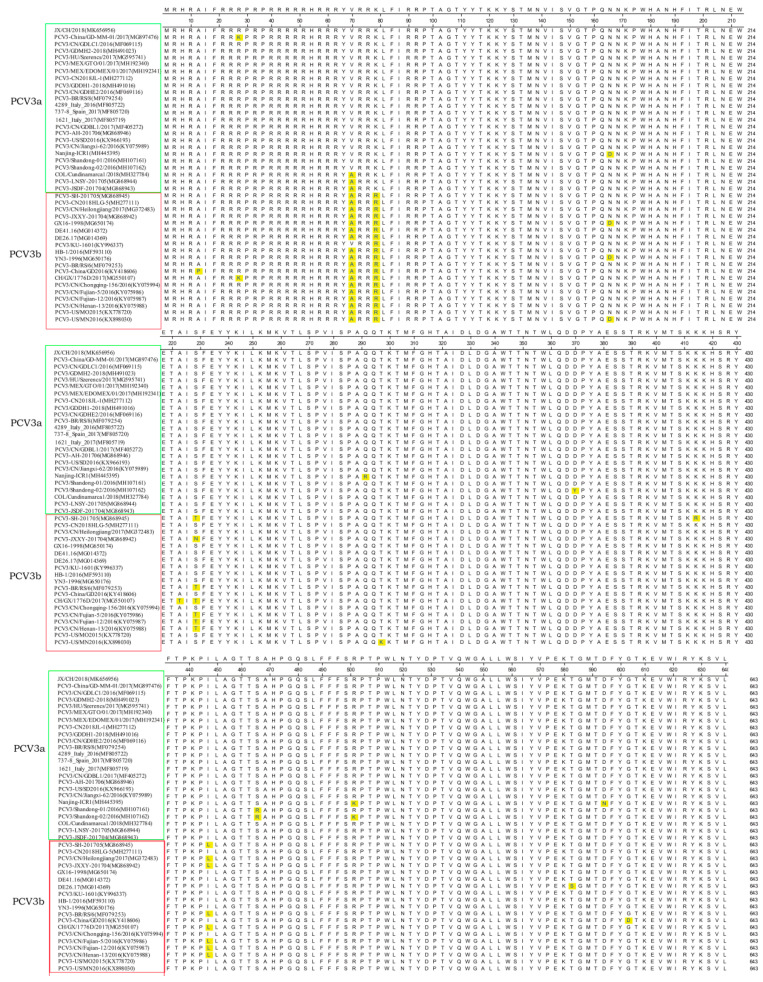
Alignment analysis of the amino acid sequences of the capsid genes between the identified PCV3 strain JX/CH/2018 and reference PCV3 strains.

To characterize the genetic features of PCV3 associated with diarrhea, the complete genome sequence of a representative PCV3 strain, designated JX/CH/2018, was determined and analyzed. The complete genome of JX/CH/2018 was 2000 bp in length (the sequence was deposited into the GenBank under the accession number of MK656956), including an encoding 296-aa Rep gene, and an encoding 224-aa Cap protein gene. A multialignment analysis indicated that JX/CH/2018 shared 97.7–99.7% nt identity at the complete genome level, and 97.1–99.8% at the nt level and 97.2–100% at the aa level of the capsid protein when compared with reference PCV3 strains retrieved from GenBank (Table S2). The Cap protein is the major structural protein of PCV3 and the major target of the host immune response. Through multiple sequence alignment of Cap protein, we discovered that the majority variant of PCV3 strains had five nucleotide mutations: T71C, A80G, C171T, G215A/C, A288G, and A448C (Figure S1) and four major amino acid mutations: A24V, R27K, S77T, and I150L (Figure 1). In the context of genetic diversity, three ORFs of PCV3 were conserved, and no insertion/deletion was observed in the same subgroup PCV3. However, several mutations were present in the capsid proteins between PCV3a and PCV3b strains—e.g., V24A, K27R (Figure 1).

**Figure 2:**
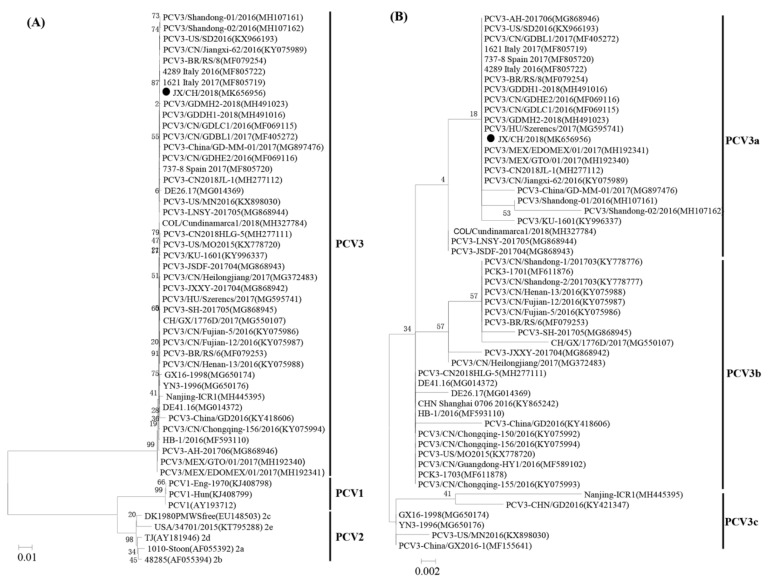
Phylogenetic trees generated based on the complete genome sequence of PCV3, PCV2, and PCV1 (**A**) and capsid protein sequences of PCV3 around the world (**B**). Bootstrap values were calculated with 1000 replicates. A bar of 0.002 indicates nucleotide or amino acid substitutions per site. “●” indicates the strain identified in this study.

A phylogenetic analysis based on the complete genomes of JX/CH/2018 determined in this study and reference strains of PCV3 indicated that JX/CH/2018 was clustered into PCV3 group, and distinct from the type 1 and type 2 porcine circoviruses (Figure 2A). Based on aa sequences of the capsid protein, the PCV3 strains could be divided into three subgroups—namely, PCV3a, PCV3b, and PCV3c (Figure 2B). JX/CH/2018 along with PCV3 strains identified in China, Mexico, Spain, Italy, and USA belonged to PCV3a subgroup.

### 3.3. Clinical Signs, Gross Lesions, and Histopathological Changes

**Figure 3:**
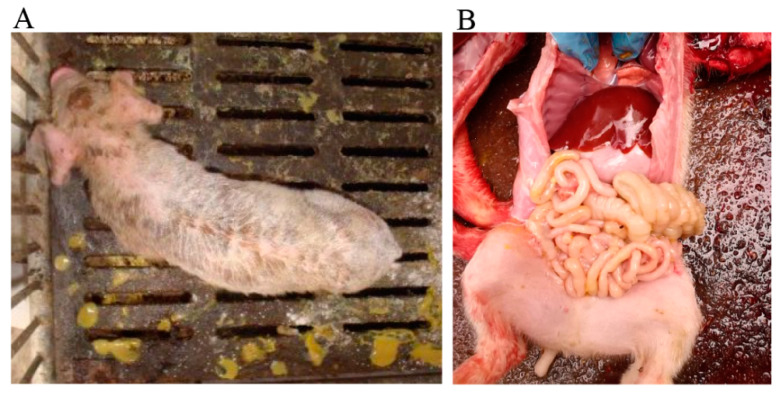
Clinical assessment (**A**) and gross lesions (**B**) of the piglets experimentally infected with PCV3.

Six 3-week-old piglets were inoculated with PCV3 sole-positive intestinal contents in this study. All six piglets exhibited typical clinical signs of diarrhea, characterized by slight dehydration, anorexia, and depression (Figure 3A). Necropsy examinations showed a markedly dilated stomach that was filled with coagulated milk, and intestines were distended with pale yellow fluid (Figure 3B).

**Figure 4:**
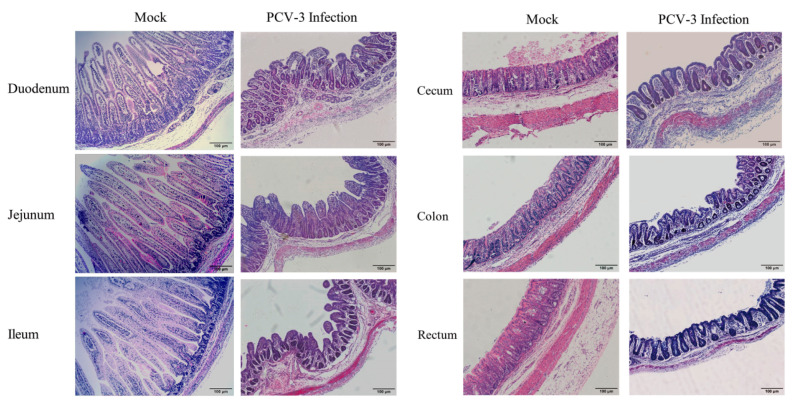
Histopathologic lesions of the small intestine induced by PCV3 strain JX/CH/2018 infection. Severe villous atrophy in the duodenum, jejunum, and ileum of the piglets under PCV3 infection are presented.

No gross lesions were observed in other organs, including colon, rectum, lung, heart, liver, kidney, spleen, and muscle. Moderate to severe atrophic villi were observed in duodenum, jejunum, and ileum in PCV3 infected piglets. The villous changes were involved in epithelial degeneration and necrosis in the entire small intestine. Microscopic lesions were not apparent in the sections of cecum, colon, and rectum (Figure 4). Villus height (VH) and crypt depth (CD) were measured, and the ratio of VH/CD was calculated and compared on intestinal sections of PCV3-infected and negative control piglets (Table 2). PCV3-infected piglets had significantly decreased average heights of villus (*p* < 0.01), and depths of crypt (*p* < 0.05). The ratio of VH/CD in duodenum, jejunum, ileum, and cecum was statistically significant (*p* < 0.01) when compared with the negative controls, but the average height of villus, depth of crypt, and the ratio of VH/CD were not significantly different in colon and rectum between the infected and negative control of piglets.

## 4. Discussion

PCV3 is a novel porcine viral pathogen that was first recognized in commercial pig farms in the USA in 2016, and then it was reported in dozens of counties, including China, South Korea, Thailand, Italy, Poland, and so on [5,6,17,24,25]. Up to date, PCV3 has been detected in pigs within different clinical presentations, including respiratory syndrome, reproductive failure, multisystemic inflammation, and gastrointestinal and neurological disorders [4,21,26]. The data regarding the association of PCV3 and diarrhea are limited. Zhai et al. described a higher prevalence of PCV3 in weaned pigs that suffered from diarrhea than those without diarrhea. [15]. In this study, we demonstrated a higher detection frequency of PCV3 in diarrheal suckling (27.28%) and weaned piglets (14.29%) than that of non-diarrheal piglets (6.67% in suckling and 4.76% in weaned piglets). This result was consistent with previous studies [13,17,20].

Generally, co-infection situations are common in diarrheal pigs affected by different etiologic agents. PCV2 and PRRSV are common agents in pigs in China, and the co-infections of these two viruses exacerbate disease severity [15]. In our study, PCV2 was frequently detected in diarrheal and non-diarrheal suckling and weaned piglets, and co-infections of PCV2 and PCV3 were identified in both diarrheal and non-diarrheal samples. Surprisingly, on one of the five pig farms in which the samples were collected, PCV3 was solely detected with a high rate in suckling piglets with severe diarrhea, and no other diarrhea associated pathogens were detectable. This fact implied that PCV3 might be a causative agent of diarrhea in pigs. However, no data are available in terms of the pathogenicity and pathogenesis of PCV3 due to the lack of isolated virus. Thus, in addition to elucidate the genetics of PCV3 responsible for pig diarrhea, clinical signs, gross lesions, and histopathological changes of pigs affected by PCV3 alone were also evaluated. Under microscopic examination, moderate to severe atrophic villi was observed in duodenum, jejunum, and ileum in PCV3-infected piglets, and the heights of villi, depths of crypt, and the ratio of VH/CD in duodenum, jejunum, ileum, and cecum in PCV3-infected piglets were significantly decreased.

To our knowledge, this is the first report on the pathogenicity induced by PCV3 associated with swine diarrhea. However, further study is needed to reveal the pathogenesis of PCV3 associated with diarrhea by a PCV3 infection/disease animal model. In previous reports, the majority of variant PCV3 strains on the capsid protein from cattle and dogs were observed at the positions of D124Y and V206A, respectively [27,28,29]. In the present study, we found that two mutations (V24A and K27R) were present when comparing PCV3a with PCV3b strains, indicating that variations existed in the field PCV3 strains. So, more molecular epidemiology investigations are needed in the future.

## 5. Conclusions

The present study demonstrated that PCV3 was widely circulating in diarrheal suckling and weaned piglets. The complete genome sequence of a representative PCV3 strain, JX/CH/2018, from a piglet with diarrhea in Jiangxi Province, China was determined and characterized. Clinical signs, gross lesions, and histopathological changes were observed in suckling piglets inoculated with PCV3. Moderate to severe atrophic villi changes were noticed in duodenum, jejunum, and ileum in PCV3 experimentally infected pigs. The average heights of villi, the depths of crypt, and the ratio of VH/CD in duodenum, jejunum, ileum, and cecum were significantly decreased in PCV3-infected piglets when compared with that of negative control piglets. The findings of this study increase the knowledge of epidemiology, viral genetics, pathogenicity, and pathogenesis of PCV3.

## Figures and Tables

**Table 1:** Detection results of Porcine circovirus 3 (PCV3) and other associated viruses in pigs with diarrhea and non-diarrhea.

State	Diarrhea		Non-Diarrhea	
Pig Stage	Suckling	Weaned	Suckling	Weaned
Sample No.	55	35	30	21
PCV3	15 (27.28%)	5 (14.29%)	2 (6.67%)	1 (4.76%)
PCV2	17 (30.91%)	12 (34.29%)	5 (16.67%)	6 (28.57%)
CSFV	0 (0)	0 (0)	0 (0)	0 (0)
PRV	0 (0)	0 (0)	0 (0)	0 (0)
PRRSV	0 (0)	0 (0)	0 (0)	0 (0)
TGEV	0 (0)	0 (0)	0 (0)	0 (0)
PoRV	0 (0)	0 (0)	0 (0)	0 (0)
PEDV	3 (5.45%)	2 (5.71%)	0 (0)	0 (0)
PDCoV	1 (1.82%)	0 (0)	0 (0)	0 (0)
SADS-CoV	0 (0)	0 (0)	0 (0)	0 (0)
PCV3 + PCV2	5 (9.09%)	2 (5.71%)	0 (0)	1 (4.76%)
PCV3 + PEDV	0 (0)	0 (0)	0 (0)	0 (0)
PCV3 + PDCoV	0 (0)	0 (0)	0 (0)	0 (0)

**Table 2:** Mean villus height, crypt depth, and villus/crypt ratio in various intestinal sections of PCV3-infected and negative control piglets.

Item	Duodenum (Mean ± SD, μm)			Jejunum (Mean ± SD, μm)			Ileum (Mean ± SD, μm)		
	PCV3-Infect	Control	*p*-Value	PCV3-Infect	Control	*p*-Value	PCV3-Infect	Control	*p*-Value
villus height	87.02 ± 13.19	287.04 ± 57.04	0.000 ***	75.74 ± 21.92	304.16 ± 82.67	0.000 ***	59.57 ± 12.51	173.55 ± 25.04	0.000 ***
Crypt depth	51.96 ± 8.72	59.42 ± 12.52	0.009 **	45.87 ± 14.84	62.59 ± 14.16	0.000 ***	37.30 ± 6.61	43.77 ± 9.29	0.007 **
Villus/Crypt ratio	1.70 ± 0.27	4.79 ± 1.08	0.000 ***	1.66 ± 0.32	4.91 ± 1.01	0.000 ***	1.61 ± 0.28	4.07 ± 0.75	0.000 ***
Item	Cecum (Mean ± SD, μm)			Colon (Mean ± SD, μm)			Rectum (Mean ± SD, μm)		
	PCV3-infect	Control	*p*-value	PCV3-infect	Control	*p*-value	PCV3-infect	Control	*p*-value
villus height	66.53 ± 7.67	74.71 ± 9.95	0.002 **	61.83 ± 9.15	63.85 ± 8.67	0.407	56.77 ± 8.38	59.11 ± 18.52	0.536
Crypt depth	31.77 ± 5.30	48.83 ± 8.29	0.000 ***	32.30 ± 7.42	29.47 ± 5.63	0.128	33.85 ± 5.00	36.60 ± 5.86	0.084
Villus/Crypt ratio	1.56 ± 0.26	2.14 ± 0.35	0.000 ***	1.99 ± 0.46	2.22 ± 0.40	0.074	1.71 ± 0.37	1.65 ± 0.58	0.635

Note: ** indicates 0.001 < *p* ≤ 0.01, *** indicates *p* ≤ 0.001.

## Supplementary Materials

The following are available online at https://www.mdpi.com/2076-2615/10/12/2324/s1, Figure S1: Alignment analysis of the nucleotide sequences of the capsid genes between the identified PCV3 strain JX/CH/2018 and PCV3 reference strains. Table S1: PCR/RT-PCR primers used in this study. Table S2: Multiple alignment analysis of nucleotide and amino acid sequence of the complete genome (A) and capsid protein (B) constructed based on the PCV3 isolated in this study and reference strains retrieved from GenBank.
