# Supplementary material for: Porcine Circovirus Type 3 in Pig Farms Experiencing Diarrhea in Jiangxi, China: Prevalence, Genome Sequence and Pathogenicity
Source: Animals (Basel). 2020 Dec 7;10(12):2324. doi: 10.3390/ani10122324 (PMC7762375; doi:10.3390/ani10122324)
Supplement: Supplementary file 1 [file animals-10-02324-s001.zip › animals-1016347-suppl-final/Supplementaly materials/animals-1016347-table s1 and figure s1.docx]

Article

Porcine Circovirus Type 3 in Pig Farms Experiencing Diarrhea in Jiangxi, China: Prevalence, Genome Sequence and Pathogenicity

Fanfan Zhang, Weifeng Yuan, Zhiquan Li, Yuhan Zhang, Xiuxiu Zeng, Min Zhao, Yu Ye, Zhen Ding, Houjun He, Qiong Wu, Deping Song and Yuxin Tang

**Table S1.** The RT-PCR primers used in this study.

| **Primer Names** | **Sequence (5′–3′)** | **Target Gene** | **Products (bp)** | **Purpose** |
| --- | --- | --- | --- | --- |
| PCV3-F | TGGTGCCGTAGAAATCTGTC | Capsid | 408 | Detection |
| PCV3-R | GCCTAAACGAATGGGAAACT |  |  |  |
| PCV3-1F | TAGTATTACCCGGCACCTCGGAACC | Genome | 1257 | Genomic sequencing |
| PCV3-1R | ACAGGTAAACGCCCTCGCATGTGGG |  |  |  |
| PCV3-2F | TGCACTTGTGTACAATTATTGCG | Genome | 1075 |  |
| PCV3-2R | ATCTTCAGGACACTCGTAGCACCAC |  |  |  |
| PCV3-qF | CACTTCATTACCCGCCTAAACG | Capsid | 170 | Detection by real-time PCR |
| PCV3-qR | GTCTTGGAGCCAAGTGTTTGTG |  |  |  |
| PCV2-F | ATGGTATGGCGGGAGGAGTA | Capsid | 248 | Detection |
| PCV2-R | GCGGTGGACATGATGAGATT |  |  |  |
| PDCoV N1-F | CCAAACGCAACCCCAACAATCC | Nucleocapsid | 329 | Detection |
| PDCoV N1-R | CTTCTCAGTGTCTGCAGAGCCG |  |  |  |
| PEDV-F | GTATTGGTGGTGAGCGGAAT | Spike | 486 | Detection |
| PEDV-R | CCTGTTCCGCCATTCTCTCA |  |  |  |
| TGEV-F | TATTTGTGGTTTTGGTTATAATGC | Spike | 886 | Detection |
| TGEV-R | GGCTGTTTGGTAACTAATTTGCCA |  |  |  |
| PoRV F | TATTCAAATATAAGTGATTTAATTCAAC | VP6 | 298 | Detection |
| PoRV-R | TAATACCTGACAGCTTTCTTAATGC |  |  |  |
| SADS-CoV-F | ACAACACGGTCCCTGTGACCGAA | Nucleocapsid | 551 | Detection |
| SADS-CoV-R | GGAACGCCCCGCACGAGTATAAA |  |  |  |
| PRV-F | CCCTGGACGCGAACGGCACGAT | gE | 502 | Detection |
| PRV-R | CTCCGAGGAGCGCAGCACCACGTGTT |  |  |  |
| CSFV-F | GACACAAGYGCAGGCAAYAG | E2 | 431 | Detection |
| CSFV-R | AGTGGGTTCCAGGARTACAT |  |  |  |
| PRRSV-F | ATGTTGGGGAAGTGCTTGACCGCGT | ORF5 | 603 | Detection |
| PRRSV-R | CTAGAGACGACCCCATTGCTCCGCT |  |  |  |


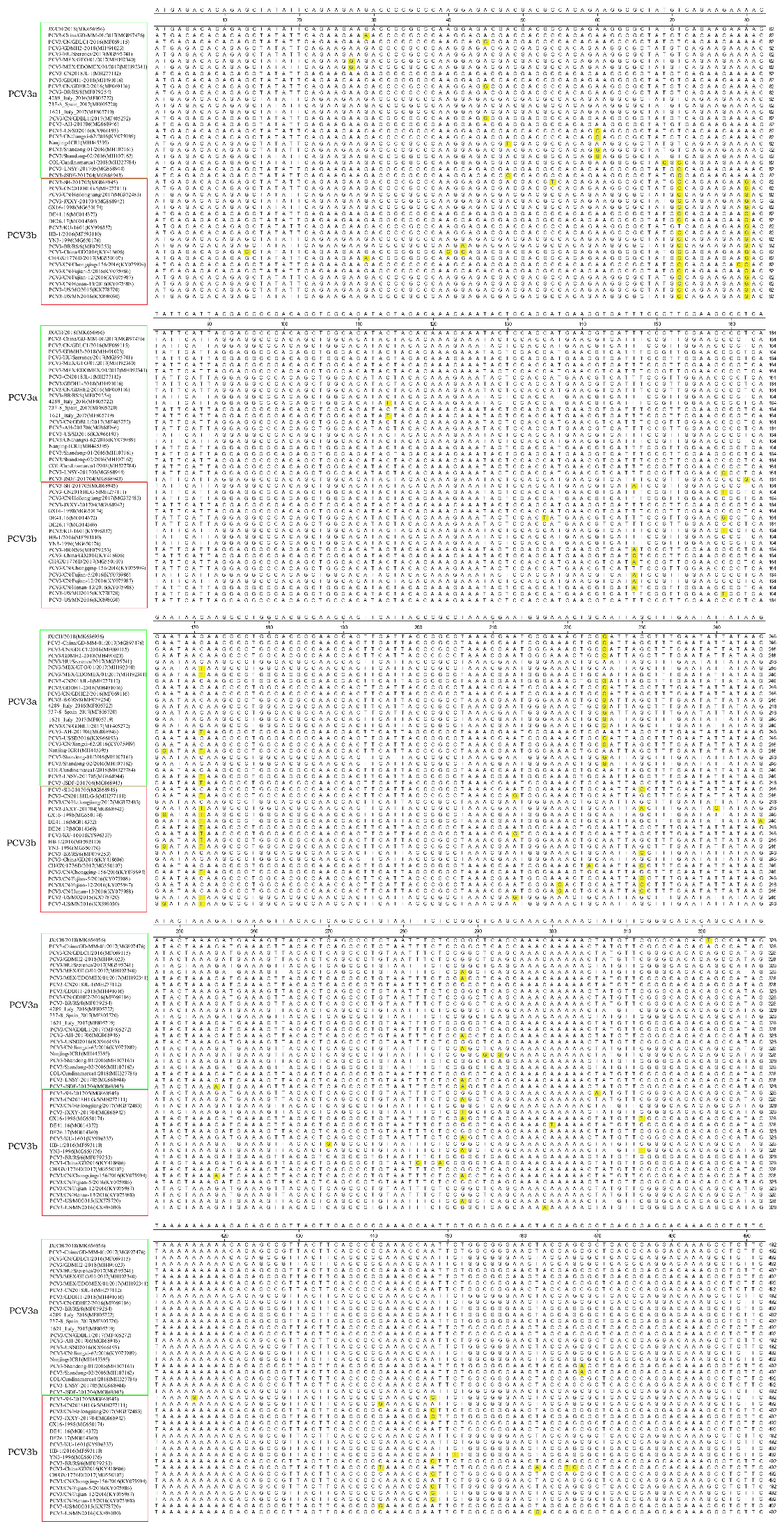


**Figure S1.** Alignment analysis of the nucleotide sequences of the capsid genes between the identified PCV3 strain JX/CH/2018 and PCV3 reference strains.

**Publisher’s** **Note:** MDPI stays neutral with regard to jurisdictional claims in published maps and institutional affiliations.

| 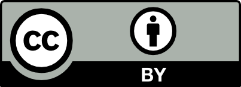 | © 2020 by the authors. Licensee MDPI, Basel, Switzerland. This article is an open access article distributed under the terms and conditions of the Creative Commons Attribution (CC BY) license (http://creativecommons.org/licenses/by/4.0/). |
| --- | --- |
